# Unravelling the Role of Melanin in Cd and Zn Tolerance and Accumulation of Three Dark Septate Endophytic Species

**DOI:** 10.3390/microorganisms8040537

**Published:** 2020-04-08

**Authors:** Charlotte Berthelot, Asfaw Zegeye, Dalia A. Gaber, Michel Chalot, Philipp Franken, Gábor M. Kovács, Corinne Leyval, Damien Blaudez

**Affiliations:** 1 Université de Lorraine, CNRS, LIEC, F-54000 Nancy, France; 2 CTIFL, Centre de Carquefou, F-44483 Carquefou, France; 3 Botany and Microbiology Department, Faculty of Sciences, Assiut University, Assiut 71515, Egypt; 4 Leibniz Institute for Vegetable and Ornamental Crops, 14979 Groβbeeren, Germany; 5 Université de Bourgogne-Franche-Comté, CNRS, Laboratoire Chrono-Environnement, F-25211 Montbéliard, France; 6 Université de Lorraine, F-54000 Nancy, France; 7 Erfurt Research Centre for Horticultural Crops, University of Applied Sciences Erfurt, 99090 Erfurt, Germany; 8 Department of Plant Anatomy, Institute of Biology, Eötvös Loránd University, 1117 Budapest, Hungary; 9 Plant Protection Institute, Centre for Agricultural Research, 1022 Budapest, Hungary

**Keywords:** *Cadophora* sp., kojic acid, *Leptodontidium* sp., *Phialophora mustea*, sulcotrione, tricyclazole

## Abstract

Dark septate endophytes (DSEs) are often trace element (TE)-tolerant fungi and are abundant in TE-polluted environments. The production of melanin, a black polymer found in cell walls, was hypothesized by several authors to play a role in the TE tolerance of DSEs. To test this hypothesis, we established a series of experiments using albino strains and melanin inhibitors and examined the responses to Cd and Zn. Six DSEs belonging to genera *Cadophora* sp., *Leptodontidium* sp. and *Phialophora mustea*, were evaluated. The strains mainly produced 1,8-dihydroxynaphthalene (DHN) melanin whereas 3,4-dihydroxyphenylalanin melanin was also synthetized. Cd and Zn decreased melanin synthesis in most of the strains. A reduction in melanin concentration in hyphae through the use of tricyclazole, an inhibitor of DHN-melanin synthesis, did not reduce the tolerance of the strains to Cd and Zn. Similarly, albino mutants of *Leptodontidium* sp. were not more sensitive to Cd and Zn than the WT strain. Moreover, tricyclazole-treated colonies accumulated less Cd but more Zn compared to untreated colonies. The Cd and Zn contents of *Leptodontidium* albino strains were variable and similar to that of the WT. The results suggest that melanin production is not an important functional trait that contributes to Cd and Zn tolerance, but might contribute to Cd accumulation.

## 1. Introduction

Dark septate endophytes (DSEs) are polyphyletic, sterile or conidial, ascomycetes characterized by darkly pigmented mycelia and septate hyphae [1]. They have been observed in the roots of more than 600 plant species spanning 100 plant families [2]. DSEs have a wide geographical distribution (e.g., arctic, boreal, alpine, temperate forest, or tropical ecosystems) and display strong abilities to tolerate a range of environmental stresses (e.g., drought, salinity, pollution) [2,3,4,5].

Regarding metal-contaminated soils, DSE colonization was reported in plant roots of Pb and/or Zn mining or smelting sites and Ni/Cu smelter areas [3,6,7]. Moreover, in these environments, the relative abundance of DSEs increased with TE stress compared to other root-associated fungi [3,6]. This tolerance towards TE stress has also been confirmed in vitro using agar-plate tests [3,8,9]. However, the current knowledge on the physiological traits underlying DSE tolerance to TE is scarce, especially at the molecular and cellular levels. Recent studies [10,11,12] suggested that DSEs could survive in TE-contaminated environments through three mechanisms: (i) extracellular chelation to prevent TE penetration into cells, (ii) complexation and sequestration of TE in cellular compartments to inhibit their toxicity, and (iii) restoration of TE-induced injuries. However, numerous studies reported the beneficial inoculation of several plant species to cope with metal toxicity [13,14,15,16,17,18]. Several authors hypothesized that the high melanin-derived pigmentation could partially explain the relatively high TE tolerance of DSEs and their TE accumulation capacities in the mycelia of DSEs [19,20].

Melanin is a common compound found in animals, plants, bacteria and fungi [21]. Fungal melanins are present in cell walls and spores, associated with protein or carbohydrates, or can be released in the extracellular fraction [22]. They are biopolymers synthesized from phenolic or indolic compounds. While they are non-essential in primary metabolic pathways, melanins may still serve essential survival functions, particularly under stressful environmental conditions such as TE-contaminated soils [21,22,23]. Indeed, carboxyl and hydroxyl groups of melanin can bind TE with a high affinity. However, the nature and content of melanin vary drastically among fungi, modifying its capacity towards TE binding. As a matter of fact, there are three main types of melanin: (i) 3,4-dihydroxyphenylalanine (DOPA)-melanin, (ii) pheomelanin, and (iii) allomelanin (including 1,8-dihydroxynaphthalene (DHN)-melanin, catechol-melanin, γ-glutaminyl-3,4-hydroxybenzene (GDHB)-melanin and pyomelanin) [22]. They are synthesized from the tyrosine precursor, with the exception of DHN-melanin, which is synthesized from malonyl- or acetyl-coA precursors. Several authors assume that their phylogeny would largely govern the type of melanin produced by fungi. For instance, basidiomycetes produce GDHB-, catechol- and DOPA-melanins, whereas ascomycetes produce DHN-, pyomelanin and sometimes DOPA-melanin [22,24]. However, recent studies reported several exceptions and knowledge about melanin biosynthesis pathways in fungi needs further detailed investigation [25]. Melanin-specific metabolic inhibitors and the use of albino mutants represent powerful strategies to decipher the properties of fungal melanin and the biosynthetic pathways that are involved [26].

The melanin biosynthetic pathways and the properties of the melanin of human pathogenic fungi led to several investigations [27]. Conversely, studies concerning melanin in DSEs are limited [19,20,28]. More generally, the relationship between melanin content and TE-tolerance and TE-accumulation in ascomycetes is ambiguous and is still under debate. For instance, a recent publication focusing on the DSE *Exophiala pisciphila* reported that melanin was necessary for Cd tolerance [20] whereas Cd tolerance was unchanged between a melanized strain of the root-rot pathogen *Gaeumannomyces graminis* and its isogenic albino strain [26]. These results suggest that melanin would not be the major factor of TE tolerance in these fungi. The same controversy emerges for the relationship between melanin content and TE accumulation by DSEs. Indeed, Gadd and Rome (1988) and Siegel et al. (1990) reported a decreased accumulation of TE in melanin-inhibited *Cladosporium* sp. whereas that was not the case for *E. pisciphila* [20,29,30]. However, whether melanin plays an important role in TE tolerance and accumulation in DSEs still needs to be demonstrated.

Therefore, in the present study, we investigated the role of melanin in Cd and Zn tolerance and accumulation in six DSE strains belonging to three genera of Helotiales that differed in their mycelial melanin concentration. In a first approach, in silico analyses and in vitro tests were performed to reveal which pathways were involved in melanin biosynthesis. Second, melanin-specific metabolic inhibitors and albino mutants of *Leptodontidium* sp. were used to reveal a putative relationship between TE tolerance and accumulation with melanin concentration in mycelia.

## 2. Materials and Methods

### 2.1. Searching for Homologous Genes

**Figure 1:**
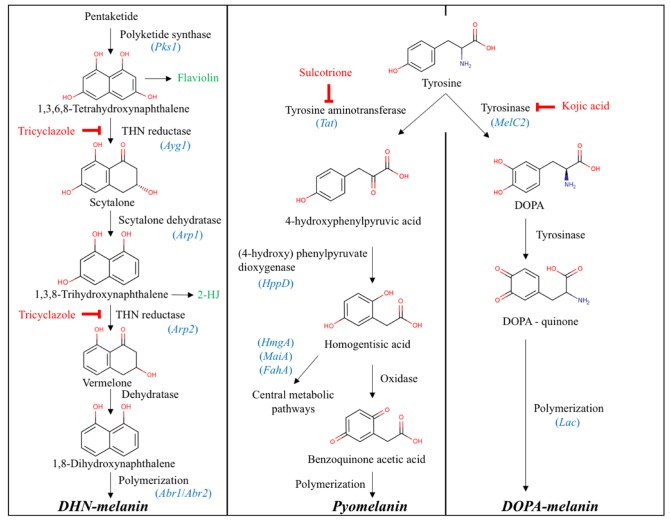
Melanin synthesis pathways occurring in *Ascomycetes*. In blue are given the genes/proteins involved in the different steps. In red are indicated the metabolic inhibitors we used. In green are given the intermediate compounds that accumulate after tricyclazole treatment. Please, refer to Tables S1 and S2 for more information on the protein IDs found in *Leptodontidium* sp. and *Cadophora* sp. DHN = 1,8-dihydroxynaphthalene; DOPA = 3,4-dihydroxyphenylalanine; 2-HJ = 2-hydroxyjuglone.

The Mycocosm JGI [31] portal for the genome of *Cadophora* sp. DSE 1049 (https://mycocosm.jgi.doe.gov/Cadsp1/Cadsp1.home.html) [28] and *Leptodontidium* sp. PMI_412 (https://mycocosm.jgi.doe.gov/Leptod1/Leptod1.home.html) genomes was used to identify the genes involved in melanin biosynthesis pathways. We followed a blastp reciprocal best hit search strategy to identify homologous proteins in these two genomes. Based on literature data [32,33], we searched for proteins involved in the biosynthesis of DHN-melanin, DOPA-melanin and pyomelanin—the three forms of melanin produced by ascomycetes (Figure 1).

When we followed the method of Tsai et al. [32], Alb1 (Pks1)—polyketide synthase AAC39471.1, Arp1—scytalone dehydratase (PF02982) AAC49843.1, Arp2—1,3,6,8-tetrahydroxynaphthalene (THN) reductase AAF03314.1, Abr1—brown 1 AAF03353.1, Ayg1—yellowish-green 1 AAF03354.1 and Abr2—brown 2 AAF03349.1 were used. We first made a blastp with the original protein sequences to the *Aspergillus fumigatus* Af293 genome [34,35] at Mycocosm JGI (https://mycocosm.jgi.doe.gov/Aspfu1/Aspfu1.home.html) and used the best hit proteins for the reciprocal best hit search on the two genomes. When we followed the method of Li et al. [36], we used the proteins involved in different melanin synthesis pathways identified in the genome *Wangiella* (*Exophilia*) *dermatitidis* based on the results of the transcriptomic analyses of *Fonsecaea monophora*. According to their results, we identified those proteins in the complete genome of *E. dermatitidis* UT8656 [37,38] at Myocosm JGI (https://mycocosm.jgi.doe.gov/Exode1/Exode1.home.html) and used those proteins for the reciprocal best hit blastp search.

### 2.2. Strains and Culture Medium

Six DSE strains were used in the present study. Strains Me07 and Pr30 belong to *Leptodontidium* sp., strains Fe06 and DSE 1049 belong to *Cadophora* sp., and strains Pr29 and Va46 belong to *Phialophora mustea*. They were isolated from metal-polluted poplar phytomanagement sites and were all described previously [9,39]. We also used five isogenic albino mutant strains of *Leptodontidium* sp. They were obtained from agrotransformation of the strain *Leptodontidium* sp. Me07 [40]. The strains were cultured on Pachlewski [41] agar medium at 24 °C in the dark.

### 2.3. Strain Sensitivity to Melanin Inhibitors and to TE

The strain sensitivity to different inhibitors of melanin biosynthesis was evaluated by investigating the minimum inhibitory concentration (MIC) of three melanin inhibitors on fungal growth. Kojic acid inhibits tyrosinase which catalyzes two steps (tyrosine oxidation to DOPA and conversion of DOPA to dopaquinone) of the DOPA-melanin pathway [19]. Tricyclazole inhibits both tetra- and trihydroxynaphthalene reductases in the DHN-melanin pathway. These enzymes catalyze the reduction of 1,3,6,8-tetrahydroxynaphthalene to scytalone and 1,3,8-trihydroxynaphthalene to vermelone, respectively [22]. Sulcotrione inhibits the p-dihydroxy-phenylpyruvate dehydrogenase which catalyzes the dehydrogenation of 4-hydroxy-phenylpyruvate to homogentisic acid in the pyomelanin pathway [42]. Plugs (6 mm) were cut from the edge of actively growing two-week-old fungal colonies and placed on solid Pachlewski medium amended with melanin inhibitors at the following concentrations: 0 to 100 μg/mL tricyclazole (Sigma-Aldrich, Saint-Quentin-Fallavier, France), 0 to 1000 μg/mL kojic acid (Sigma-Aldrich), and 0 to 200 μg/mL sulcotrione (Sigma-Aldrich). TE tolerance tests were performed with or without tricyclazole and 0 to 1.5 mM CdCl_2_ or 0 to 9 mM ZnCl_2_. The TE tolerance of isogenic albino was also evaluated with the same range of Cd and Zn concentrations. For all treatments, diameter of colony, dry biomass and mycelial pigmentation were recorded after two weeks of incubation at 24 °C.

### 2.4. Effect of Melanin Inhibitors and TE on Mycelial Melanin Concentration

The quantification of melanin concentration in mycelia was performed with fungal strains cultured on Pachlewski agar media covered with sterile cellophane sheets and amended with or without TE and melanin synthesis inhibitors. Tricyclazole (10 μg/mL) and kojic acid (50 μg/mL) were used separately or in combination in the culture medium. Sulcotrione was omitted in this experiment because it had no impact on strain pigmentation. TE were used as follows: ZnCl_2_ at 1 mM and CdCl_2_ at 200 μM. Mycelia were harvested after two weeks of growth. Samples were dried overnight at 60 °C, weighed and ground into powder.

To determine the melanin concentration in mycelia, an extraction protocol was carried out according to a modified method of Zou et al. (2010) [43]. The powder samples (1 g) were washed with 30 mL of distilled water for 5 min, followed by a centrifugation step at 4000 rpm for 5 min. The pellets were resuspended with NaOH 6 M to reach a final pH of 12. After a sonication step of 24 h at 50 °C and 40 Hz, the samples were centrifuged at 4000 rpm for 5 min. The extracts were further acidified to pH 2 with 8 M HCl to precipitate melanin, followed by a centrifugation step at 10,000 rpm for 20 min. Two washes of melanin with chloroform and ethyl acetate were finally performed. For quantification, melanin was dissolved in 0.1 M NaOH and the OD_400nm_ of the solution was determined. Blanks consisted of 0.1 M NaOH. A standard curve was obtained with melanin from *Sepia officinalis* (Sigma-Aldrich).

### 2.5. Quantification of TE in Mycelia

To measure the concentrations of TE in mycelia, dry biomass (100 mg) was digested by HNO_3_ in a MARS 5 (CEM^®^, Saclay, France) microwave oven with the following program: 15 min at 170 °C and 20 bars followed by 30 min cooling. The solutions were analyzed by inductively coupled plasma–atomic emission spectroscopy (Varian, Victoria, Australia). Two tobacco (INCT OBTL5 ichtj, Warsow, Poland) and ryegrass (CD281 O204, ERM^®^, Geel, Belgium) standard references were submitted to the same digestion procedure and analysed as part of the quality control of the protocol.

### 2.6. Fourier Transformed Infrared Resonance (FTIR) Analysis

Analysis of the Fourier transformed infrared resonance (FTIR) of melanin was performed with *Leptodontidium* sp. Me07 cultured on Pachlewski agar media covered with sterile cellophane sheets and amended with or without melanin synthesis inhibitors. Tricyclazole (10 μg/mL) and kojic acid (50 μg/mL) were used separately or in combination in the culture medium. The melanin samples were mixed with KBr (1:100 w/w) and gently homogenized in an agate mortar. The mixture of melanin and KBr was pressed into tablets by using an hydraulic press. The spectral information was collected at a resolution of 4 cm^−1^ using a Brucker Vector 22 FTIR spectrometer in the wavenumber region of 700–3700 cm^−1^.

### 2.7. Statistical Analyses

Statistical analyses were performed with R 3.1.3 (R Core Team2013) software. The normality of the data was tested using a Shapiro–Wilk test, while homoscedasticity was tested using a Fisher test. Data were then analyzed by one-way ANOVA or by a one-way non-parametric Kruskal–Wallis test (alpha level of 0.05). Pearson correlations were used to evaluate the relationship between the different parameters measured. All values reported are means ± standard errors (SE). For percentage values, we used the *arcsine* of the square root of the data for statistical analyses.

## 3. Results

### 3.1. Enzymes Involved in Melanin Biosynthetic Pathways

We identified melanin biosynthesis proteins from *Cadophora* sp. and *Leptodontidium* sp. genomes by a blastp reciprocal best hit search with *Wangiella* (*Exophilia*) *dermatitidis* and *Aspergillus fumigatus* protein sequences [32,36]. The identified proteins from the two genomes of DSE fungi are shown in Table S1 and Table S2. The reciprocal best hit identified homologous proteins in case of four enzymes (Ayg1, Alb1, Arp1, Arp2); nevertheless, the other two (Abr1, Abr2) were also identified in both *Cadophora* sp. and *Leptodontidium* sp. genomes when *A. fumigatus* enzyme sequences were used (Table S1). We identified at least one homologous protein of all the enzymes (Figure 1) listed in Li et al. (2016) as being involved in melanin synthesis [36]. In the case of Ayg1, Alb1 (Pks1) and Arp2, the reciprocal best hit blast using *E. dermatitidis* proteins resulted the same homologous proteins as with *A. fumigatus*. In the case of Abr2, two of the same proteins were found but those gave reciprocal best hit with *E. dermatitidis* (Table S2). Nevertheless, there was no reciprocal best hit for all the 26 proteins listed identified in *E. dermatitidis* based on the transcriptomic analyses of *Fonsecaea monophora* in the two DSE genomes and the blast results showed the same pattern, except in one laccase gene, where *Leptodontidium* sp. had homologous protein and *Cadophora* sp. did not (Table S2).

### 3.2. Effect of Biochemical Inhibitors on Melanin Accumulation

In order to reveal the main forms of melanin synthesized in the mycelia of the six DSE strains, we first performed a qualitative experiment in which each strain was grown in the presence of different melanin synthesis inhibitors (sulcotrione, tricyclazole and kojic acid), each specific of one of the three main forms of melanin commonly identified in ascomycetes. In the presence of sulcotrione, neither growth inhibition nor pigmentation loss was observed for all the strains tested (data not shown). In the presence of tricyclazole, fungal growth was only inhibited at the highest concentration tested (100 μg/mL; data not shown); but whatever tricyclazole concentration had been used, the grey/dark phenotype of the colonies of the six strains was always less pronounced when compared to the non-treated controls. Instead, we noted a reddish/brown pigmentation of the colonies, but also the medium surrounding the colonies. The diameter of the red/brown halo enlarged with increasing concentrations of tricyclazole. In the presence of kojic acid, no growth inhibition was reported. The pigmentation of strains Pr30, Me07 and Pr29 remained unchanged. Conversely, the intensity of pigmentation of Fe06, DSE 1049, and Va46 colonies was slightly reduced at 100 and 200 μg/mL kojic acid (data not shown).

**Figure 2:**
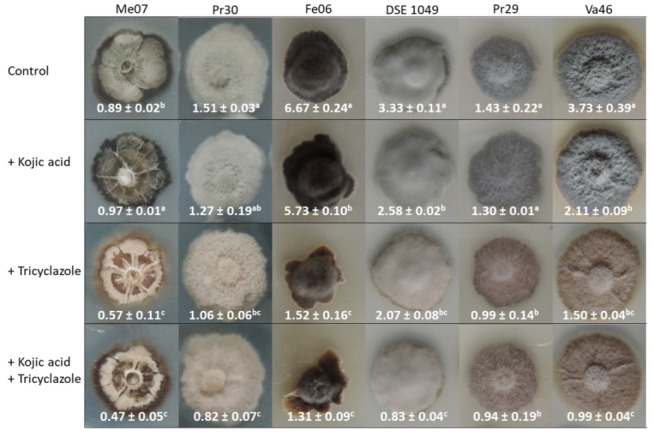
Accumulation of melanin in six DSE strains under control conditions or in the presence of inhibitors of melanin synthesis. DSEs were grown for two weeks on control Pachlewski medium or on the same medium amended with either 50 μg/mL kojic acid or 10 μg/mL tricyclazole, or a mixture of both inhibitors. A representative colony is shown and the experiment was carried out six times with similar results. Values represent melanin concentrations (mg/g DW) and are means ± SE. Significant differences between treatments (*p* < 0.05, Kruskal–Wallis test) are indicated by different letters.

Because tricyclazole and kojic acid addition induced a variation in pigmentation of the colonies in the first qualitative experiment, we sought to confirm these observations by performing in a second approach melanin quantification from mycelia grown on agar plates amended with or without 50 μg/mL kojic acid, 10 μg/mL tricyclazole or a combination of both compounds (Figure 2). The second experiment led to the same color phenotypes observed in the first one. Except for a slight decrease for Pr30 in the presence of kojic acid, growth of the strains was not affected (Table S3). Kojic acid reduced melanin concentration of Fe06, DSE 1049, and Va46 by 14%, 23%, and 44%, respectively (Figure 2). However, it did not significantly affect the melanin concentration of the three other strains. Tricyclazole inhibited the accumulation of melanin more than kojic acid in all strains. The most substantial reduction in melanin concentration was observed for Fe06 and Va46 (−77% and −60%, respectively). Moreover, the combination of both inhibitors induced the strongest reduction in melanin concentration. However, there was no significant difference between the tricyclazole treatment and the combinatory treatment (Figure 2).

### 3.3. Fourier Transform Infrared Spectroscopy (FTIR) of Melanin from Leptodontidium sp. Me07

We further tested the hypothesis of whether melanin extracted from colonies grown in the presence of the different inhibitors possessed the same characteristics. For this purpose, a detailed comparative analysis of the infrared spectra was conducted which allowed distinguishing the dominant functional groups in the presence of tricyclazole and/or kojic acid in the culture medium.

**Figure 3:**
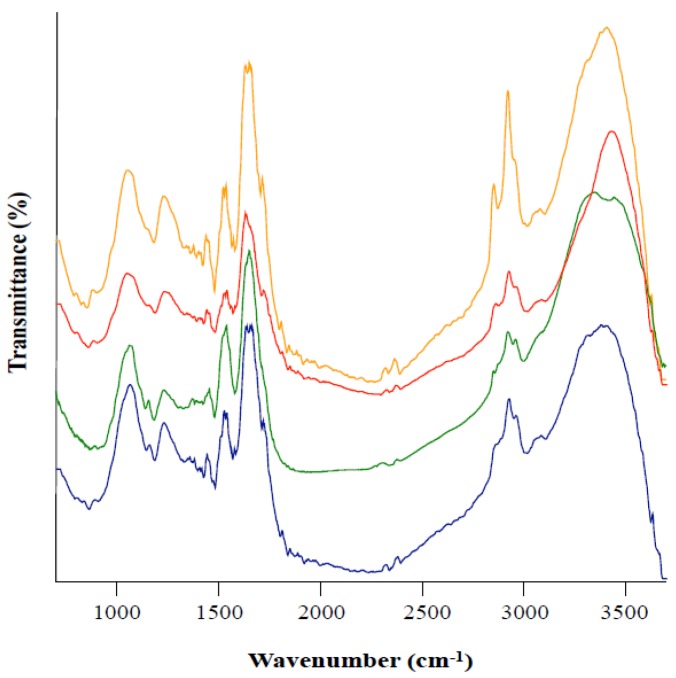
FTIR spectra of melanin extracted from Leptodontidium sp. Me07 mycelia. The mycelia were grown on control Pachlewski medium (orange) or on the same medium amended with tricyclazole (red), kojic acid (blue) or tricyclazole and kojic acid (green). Representative spectra are shown and the experiment was repeated twice (biological replicates) with similar results.

A broad band at the 3500–3200 cm^−1^ region is characteristic of the stretching vibration of N-H and O-H groups. Bands in the area of 3000–2800 cm^−1^ are assigned to the stretching of C-H groups typical of aliphatic organic compounds (Figure 3). The band/shoulder at ≈ 1716 cm^−1^ is attributed to C=0, and the band at ≈1640 cm^−1^ is related to an aromatic ring, C=C and/or COO^-^ groups. The band around 1530 cm^−1^ can be due to N-H bending while the band at 1430 cm^−1^ corresponds to CH_2_-CH_3_ bending. The peak at ≈1230 cm^−1^ relates to phenolic C-OH stretching. The peak at ≈1045 cm^−1^ is indicative of C-H in aliphatic structure (Figure 3). In summary, the different treatments displayed an IR signature which is in agreement with previously reported melanin feature [20]. Moreover, results displayed by the different IR spectra were very similar, suggesting that melanin extracted from both the treated and non-treated colonies shared the same main features.

### 3.4. Melanin Accumulation in the Presence of Cd and Zn

**Figure 4:**
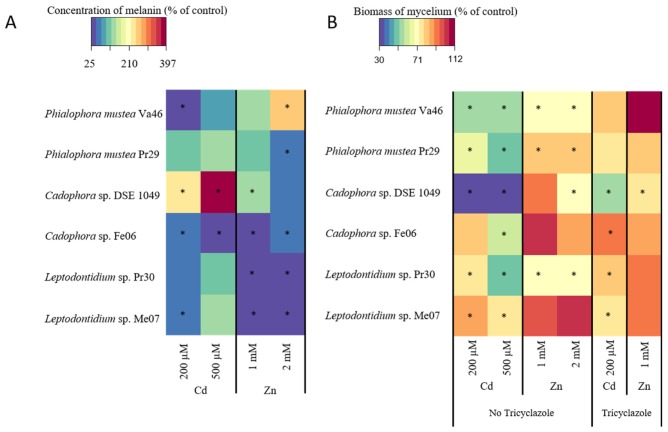
Effect of Cd and Zn on melanin accumulation and tolerance of six DSE strains. Relative hyphal melanin concentration (**A**) and relative biomass of DSE mycelia (**B**) in the presence of Cd or Zn in comparison with the control treatment (no TE added). Data are the means of *n* = 9. The control value of each strain was set to 100%. Within a given DSE strain, significant differences with the respective control treatment are denoted by asterisks (Kruskal–Wallis, *p* < 0.05).

We first investigated whether Cd and Zn had an impact on melanin accumulation by the six DSE strains. The two concentrations of Cd and Zn were chosen from preliminary experiments to produce moderate metallic stress. Except for Va46 and DSE 1049 for which 2 mM Zn and both concentrations of Cd, respectively, induced a higher accumulation of melanin, no variation or a lower concentration of melanin in hyphae was found in all other TE/strain combinations (Figure 4A). Fe06, Pr30 and Me07 strains displayed the highest loss of melanin under Zn treatment, whereas Va46 and Fe06 strains exhibited a notable decrease of melanin concentration under Cd treatment. However, analyses of Pearson correlations indicated a non-significant correlation between the melanin accumulation of the different strains and exposure to both Cd (*p* = 0.30; *r*^2^ = 0.26) and Zn (*p* = 0.72; *r*^2^ = 0.09).

### 3.5. Relationship between Melanin Accumulation and TE Tolerance

We also assessed the tolerance of the different strains by measuring the dry biomass of colonies under the same TE treatments (Figure 4B). In the absence of tricyclazole, the growth of Va46, Pr29 and Pr30 were significantly reduced from 25% to 51% and 20% to 31% for the Cd and Zn treatments, respectively. The growth of the strain DSE 1049 was more impacted by Cd (less than 30% to 35% of residual growth at both Cd concentrations). Conversely, the growth of strains Fe06 and Me07 was not significantly affected by Zn and only moderately reduced in the presence of Cd (39% and 22% for Fe06 and Me07 at 500 μM Cd, respectively).

In the presence of tricyclazole, the growth of DSE 1049 was moderately reduced under 1 mM Zn (25%) whereas a similar finding was observed for DSE 1049, Pr30 and Me07 exposed to 200 μM Cd (43%, 20%, and 23%, respectively). However, and except for Me07 at 200 μM Cd and DSE 1049 at 1 mM Zn, whatever the strain tested, growth inhibition by Cd and Zn was less pronounced under tricyclazole treatment when compared to the absence of the inhibitor.

**Figure 5:**
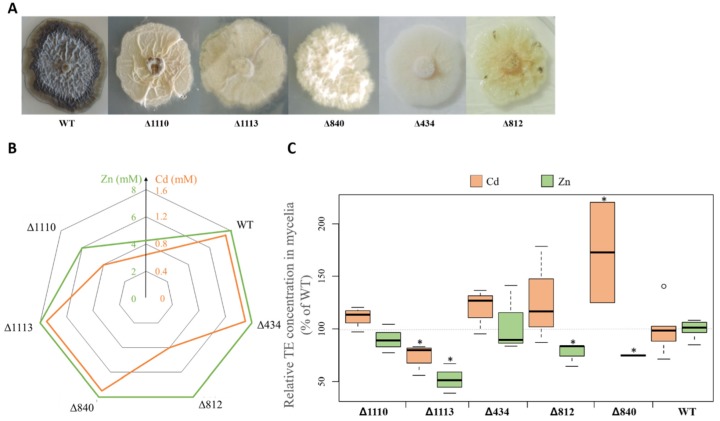
Zn and Cd tolerance and accumulation in the mycelia of *Leptodontidium* sp. Me07 albino mutants. (**A**) Phenotypes of two-week-old colonies of WT and albino mutants on Pachlewski medium. (**B**) Zn and Cd tolerance of WT and albino strains. Data represent MIC values. MIC is the minimum inhibitory concentration of metal that completely inhibited fungal growth. The experiments were repeated three times with similar results. (**C**) Cd and Zn accumulation in WT and albino mutants. Values are medians ± quartiles (*n* = 6). Cultures were incubated for 2 weeks in the presence or absence of 200 μM Cd or 1 mM Zn. Stars denote significant differences between albino strains and the WT control (*p* < 0.05, Kruskal–Wallis test).

We also investigated the putative existence of a relationship between melanin accumulation and TE tolerance by comparing the melanin-producing reference wild-type (WT) *Leptodontidium* sp. Me07 strain with isogenic albino mutant strains (Figure 5). The albino strains were obtained by random insertion of T-DNA after agrotransformation [40]. The strains all showed a white or cream phenotype (Figure 5A). To investigate whether any melanin was still produced by the albino strains, we attempted to extract melanin from these strains. A minimal quantity of powder (less than 5% of that recovered for the WT) was obtained following the same melanin extraction protocol. Moreover, we chose one sample and analyzed it by FTIR. The results revealed that the spectra from this sample were different from those obtained from typical melanin (data not shown) and indicate the absence of melanin in the albino mutants.

TE tolerance (expressed as MIC values) was generally not different between the WT and albino strains (Figure 5B). When compared to that of the WT, the MIC value for Zn was, however, lower for strain Δ1110. A similar finding was also observed for strains Δ812 and Δ1110 for Cd tolerance (Figure 5B). However, Pearson correlation analysis showed no significant relationship between melanin concentration and Cd and Zn tolerance within the isogenic mutants (Zn: *p* = 0.89; Cd, *p* = 0.92).

### 3.6. Relationship between Mycelial Melanin Concentration and TE Accumulation

**Figure 6:**
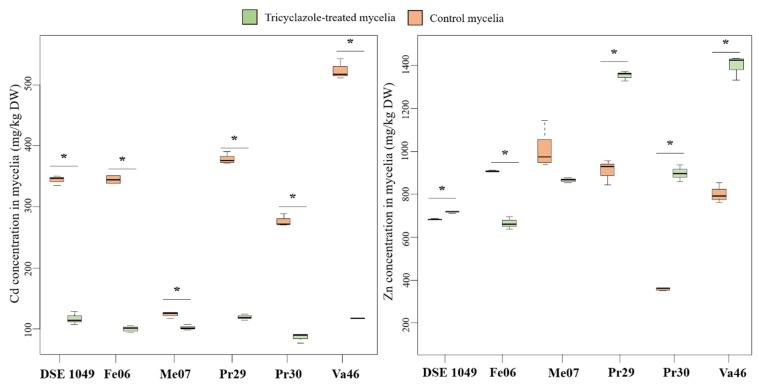
Concentration of Cd and Zn in control and tricyclazole-treated mycelia of six DSE strains. Values are medians ± quartiles (*n* = 4). Cultures were performed in Pachlewski medium for two weeks and exposed or not to 200 μM Cd or 1 mM Zn. Significant differences between control and tricyclazole treated-mycelia are denoted by stars (*p* < 0.05, Kruskal–Wallis test).

We further tested the hypothesis of the existence of a relationship between melanin concentration in hyphae and Cd and Zn accumulation. For this purpose, we first analyzed the accumulation of Cd and Zn by both tricyclazole-treated or non-treated mycelia (Figure 6). Zn content was significantly higher in tricyclazole-treated mycelia of four strains (DSE 1049, Pr29, Pr30 and Va46) when compared to non-treated mycelia (Figure 6). Conversely, Zn concentration was significantly reduced in tricyclazole-treated mycelia of Fe06. Whatever the strain, Cd concentration was lower in tricyclazole-treated mycelia when compared to non-treated mycelia (Figure 6). It decreased moderately by 17% for Me07. However, Cd concentration was more markedly lowered for the five other strains. It was indeed reduced between 66% (DSE 1049) and 78% (Va46).

We also benefited from using the albino mutants from Me07 to compare the accumulation of Cd and Zn in melanin-lacking colonies with those from the WT (Figure 5C). The accumulation of both TE varied among the albino strains. The mutants Δ434 and Δ1110 had similar concentrations of both TE when compared to the WT. Conversely, the mutant Δ840 significantly accumulated more Cd (+72%) than the WT. The opposite was found for the mutant Δ1113 (−27%). Moreover, three mutants (Δ812, Δ840 and Δ1113) accumulated significantly less Zn (23%, 25%, and 48%, respectively) compared to the WT, whereas Zn concentration was similar between the WT and the two other mutants (Figure 5C).

## 4. Discussion

### 4.1. Melanin Biosynthetic Pathways in Leptodontidium sp., Cadophora sp., and P. mustea

The present study investigated melanin biosynthesis in six DSE strains belonging to *Leptodontidium* sp., *Cadophora* sp. and *P. mustea*. In silico studies were first performed to identify the homologue putative genes/proteins involved in the biosynthetic pathways of melanin. We focused our study on the analysis of *Leptodontidium* sp. and *Cadophora* sp. genomes. We considered three melanin-specific biosynthetic pathways (pyomelanin, DHN- and DOPA-melanin) that have been already identified in Ascomycota [22]. The enzymes catalyzing the different steps of melanin synthesis, as well as the corresponding encoding genes from *A. fumigatus* and *E. dermatitidis* were previously identified [32,36]. The presence of orthologous genes encoding enzymes of the three pathways were identified from the genomes of both *Leptodontidium* sp., and *Cadophora* sp.. In relation, Knapp et al. (2018) reported a high number of melanin synthesis related genes in *Cadophora* sp. [28]. Moreover, in comparison with 34 fungi, including pathogens, saprophytes, endophytes and mycorrhizal fungi, several protein hits of *Arp1* (encoding cytalone dehydratase), *Arp2* (encoding THN reductase) and *Abr1–2* were over-represented in *Cadophora* sp. [28]. Altogether, these results suggest that *Leptodontidium* sp. and *Cadophora* sp. have the genomic potential to synthesize the three different forms of melanin.

Several authors reported that particular enzymes from the melanin pathways are not melanin-specific. Instead, they could be involved in the synthesis of numerous polyketides as well as in a large class of secondary metabolites, including melanin [44,45]. Hence, although the in silico study provided essential data, these results needed to be completed by additional information. For this purpose, we used a pharmacological approach to investigate which melanin synthesis pathway was dominant in the three DSE species we studied. Three specific metabolic inhibitors were consequently used (kojic acid, tricyclazole and sulcotrione) and melanin concentration was determined. Extracted compounds displayed the same characteristics as those from synthetic melanin. The black extracted compounds were indeed bleached in the presence of H_2_O_2_, precipitated with FeCl_3_ and did not solubilize in water and organic solvents but solubilized in NaOH [19]. FTIR analyses showed typical spectra of melanin similar to that reported for the melanin extracted from *E. pisciphila* [20]. We extracted from 0.9 (Me07) to 6.7 (Fe06) mg of melanin per gram of dry mycelium. These data are therefore consistent with previous melanin quantification in the DSE *Gaeumannomyces cylindrosporus* (mean value of 1.8 mg/g DW) and *E. pisciphila* (9.1 mg/g DW) [8,19].

In our study, sulcotrione did not affect melanin concentration for the six strains we studied, suggesting that pyomelanin is either absent or is a minor type of melanin in the three DSE species. Low concentrations of tricyclazole were highly effective in reducing the amount of melanin. High concentrations of tricyclazole (100 µg/mL) inhibited fungal growth. Likewise, previous studies reported toxicity values ranging from 20 (*E. pisciphila*) to 216 µg/mL (*Magnaporthe oryzae*) [20,46]. Generally, it is accepted that ascomycetous fungi produce allomelanin from the DHN-melanin pathway, whereas basidiomycetous fungi produce GDHB- and DOPA-melanin [22]. However, our results show that kojic acid also significantly reduced the black pigmentation of *Cadophora* sp. and *P. mustea*. Moreover, when both tricyclazole and kojic acid were used simultaneously, melanin content was significantly reduced compared to the individual inhibitory treatments. rrFTIR analyses reported the presence of melanin both in tricyclazole- and kojic-treated mycelia of *Leptodontidium* sp. Me07. Taken together, these observations suggest that these two DSE species could also produce melanin through the DOPA-melanin pathway.

The activity of several melanin pathways was previously described for several fungi [47,48]. For instance, *A. fumigatus* was reported to synthesize pyomelanin and DHN-melanin whereas *A. bisporus* produces both DOPA- and GDHB-melanin. In our study, the use of both inhibitors did not completely abolish melanin production. These results suggest either that another type of melanin was synthesized (e.g., catechol, GDHB), or that melanin precursors such as flaviolin, 2-hydroxyjuglone, 3-hydroxyjuglone accumulated following the addition of tricyclazole and absorbed at the same wavelength as melanin. Red/brown pigments accumulated in mycelia and also diffused in the agar medium in the tricyclazole treatment. Further experiments will be needed to determine the nature of these pigments. It is also possible that another type of melanin (e.g., catechol, pheomelanin) could be specifically produced by spores as suggested previously [49,50].

In conclusion, our data suggest that both DHN- and DOPA-melanin could co-occur and that DHN-melanin is the main pathway for melanin synthesis in *Cadophora* sp. and *P. mustea*. The DSE *E. pisciphila* also produced DHN-melanin [19]. The production of DOPA-melanin by *Leptodontidium* sp. remains, however, to be demonstrated. The non-inhibitory effect of kojic acid might be explained by a too low proportion of DOPA-melanin over the total pool of melanin. Altogether, the present data further extend the knowledge of melanin production by other DSE species of this environmentally relevant group of highly melanized fungi.

### 4.2. Melanin Content and TE Tolerance

**Figure 7:**
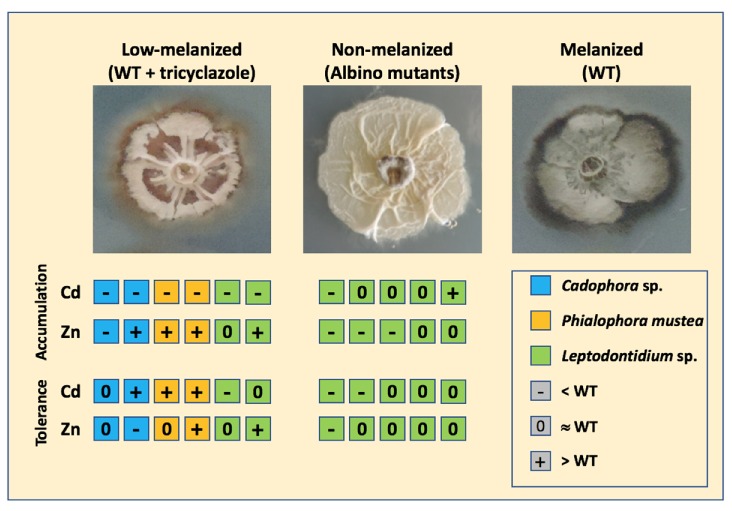
Summary of metal accumulation and tolerance as a function of melanization in DSEs. Both Cd and Zn accumulation and tolerance levels of the tricyclazole-treated and albino strains are shown relatively to those of the wild-type strain. Each square denotes a given strain. Tricyclazole was used to inhibit DHN-melanin, the major form of melanin of the three DSE species.

The accumulation of melanin in cell walls is common among fungi. It has been suggested that this function could be essential to survive under TE-stress conditions [8,22,23], but experimental confirmation is still lacking. To investigate this hypothesis, we adopted two experimental strategies. We first evaluated the level of TE tolerance for six DSE strains under DHN-melanin inhibiting conditions. This pathway was particularly targeted because it represents the main pathway of melanin in the fungal species we studied. The concentration of tricyclazole that we used significantly decreased melanin content in mycelia but did not affect fungal growth. Most of the tricyclazole-treated strains did not show a decreased tolerance towards Zn and Cd, when compared to the untreated controls (Figure 4 and Figure 7). These results suggest that the tolerance level to Cd and Zn is not melanin-dependent.

We further confirmed this finding in *Leptodontidium* sp. by studying the tolerance level to Cd and Zn of strains lacking melanin (Figure 5 and Figure 7). Generally, both Cd and Zn tolerance levels were the same between albino strains and their isogenic wild-type strain. Frederick et al. (1999) also reported that the wild-type pigmented strain and their isogenic albino mutants of *G. graminis* had the same tolerance to Cu and Cd [26]. Likewise, comparisons of Ni and Co tolerance between pigmented and non-pigmented strains of *Aspergillus nidulans* lead to the same conclusions [51,52]. Taken together, these results provide direct evidence that melanin is not the primary mechanism of TE tolerance in *Leptodontidium* sp., *Cadophora* sp. and *P. mustea*. Other intra- or extra-cellular mechanisms must occur, including either non-enzymatic or enzymatic efficient detoxifying processes against TE-induced ROS, as reported for *E. pisciphila* and *G. cylindrosporus* [8,53]. Moreover, efflux transport systems, sequestration into vacuoles, or intracellular chelation usually represent the major components of metal tolerance in other fungi [54,55,56,57]. Such mechanisms have also been proposed from the transcriptomic data of mycelia exposed to TE in the DSE fungi *E. pisciphila* and *Cadophora finlandica* [8,10,53]. However, the identification and the functional characterization of the genes/proteins that are involved have not yet been performed [12].

### 4.3. Melanin Content and TE Accumulation

Numerous studies reported the ability of melanin to bind different metal ions [23,58]. We showed, in the present study, that the six studied strains mainly produced DHN-melanin. The monomer unit contains two hydroxyl radicals that could bind metal cations, and free hydroxyl radicals are still present in the cross-linked DHN polymer. In order to evaluate the role of melanin in TE-accumulation by DHN-producing DSE fungi, we compared Cd and Zn accumulation between tricyclazole-treated or untreated mycelia. In agreement with previous studies [30,59], we report (Figure 6 and Figure 7) that the accumulation of Cd was more important by non-treated mycelia compared to tricyclazole-treated mycelia. Conversely, Zn accumulated more in tricyclazole-treated strains when compared to the non-treated strains. This observation was previously reported for Cu in *G. graminis*. In this study, the authors hypothesized that melanin precursors such as flaviolin, 2-hydroxyjuglone and 3-hydroxyjuglone could bind TE [60]. In our research, tricyclazole treatments induced the accumulation of red/brown pigments that could be responsible for the high Zn accumulation by mycelia. The variability of TE biosorption between strains could be due to the preferential affinity of melanin binding sites and to competition for the fixation sites [23,58].

However, melanin only represents a minor proportion of fungal biomass (0–5% of the mass of cell walls) compared to chitin, chitosans and glucans which dominate (respectively 8–39%, 5–33%, and 6–56%) [30]. These latter compounds are also well known in TE adsorption and could participate in the reduction of TE influx into cells [55,61]. For instance, Lanfranco et al. (2002) reported a significant increase in the content of chitin by an ericoid mycorrhizal fungus when exposed to TE [62]. Although melanin represents a small proportion of the total fungal biomass, the different accumulation patterns observed for Cd between tricyclazole- and untreated-mycelia suggest that melanin content could be a significant player in Cd adsorption by cell walls. However, the data obtained with the *Leptodontidium* sp. albino mutants did not confirm this hypothesis. Indeed, albino mycelia exhibited a different Cd accumulation pattern compared to that observed for the tricyclazole-treated mycelia (Figure 7) suggesting that the lower Cd accumulation found under tricyclazole treatment could result from an indirect effect of this compound. However, further analyses with albino mutants from other species, including *Cadophora* sp. and *P. mustea*, will be needed to confirm whether Cd accumulation is dependent of melanin in DSEs.

## 5. Conclusion

We have shown that the three DSE *Leptodontidium* sp., *Cadophora* sp. and *P. mustea* mainly produced 1,8-dihydroxynaphthalene (DHN) melanin and *Cadophora* sp. and *P. mustea* could also produce DOPA-melanin. Based on the use of albino strains of *Leptodontidium* sp. and on the application of tricyclazole, we conclude that melanin did not provide protection against Cd and Zn to these three DSE species (Figure 7). A similar conclusion can be drawn for melanization and Cd/Zn accumulation by hyphae. We focused on Cd and Zn, since they are two major TE contaminants in the environment. It will be engaging in the future to decipher whether similar conclusions could be drawn with other TE, such as Cu, As, and Ni. We speculate that the tolerance to Cd and Zn was achieved through other mechanisms such as intracellular chelation or sequestration into vacuoles. However, to reveal the detailed mechanisms involved in Cd and Zn tolerance in these DSE species, further studies will be needed.

## Supplementary Materials

The following are available online at https://www.mdpi.com/2076-2607/8/4/537/s1, Table S1: Homologous proteins (with JGI ID) involved in DHN melanin synthesis identified in the genomes of *Leptodontidium* sp. PMI_412 and *Cadophora* sp. DSE 1049 using proteins of *A. fumigatus* Af293 identified by blastp of the proteins presented by Tsai et al. (1999); Table S2: Homologous proteins (with JGI ID) involved in melanin synthesis pathways identified in the genomes of *Leptodontidium* sp. PMI_412 and *Cadophora* sp. DSE1049 using proteins of *E. dermatitidis* UT8656 following Li et al. (2016); Table S3: Effect of tricyclazole and kojic acid on fungal growth.
