# Supplementary material for: Unravelling the Role of Melanin in Cd and Zn Tolerance and Accumulation of Three Dark Septate Endophytic Species
Source: Microorganisms. 2020 Apr 8;8(4):537. doi: 10.3390/microorganisms8040537 (PMC7232325; doi:10.3390/microorganisms8040537)
Supplement: Supplementary file 1 [file microorganisms-08-00537-s001.pdf]

Table S1. Homologous proteins (with JGI ID) involved in DHN melanin synthesis identified in the genomes of *Leptodontium* sp. PMI_412 and *Cadophora* sp. DSE1049 using proteins of *Aspergillus fumigatus* Af293 identified by blastp of the proteins presented by Tsai et al. (1999).

**DHN–melanin pathway**	***Aspergillus fumigatus***			
	**Tsai et al. (1999) ID**	**Af293 jgi|ID**	***Leptodontium* sp. PMI_412**	***Cadophora* sp. DSE1049**
Ayg1	AAF03354.1	Aspfu1|3227	Leptod1|423695	Cadsp1|497711
Alb1	AAC39471.1	Aspfu1|3230	Leptod1|526612	Cadsp1|606172
Arp1	AAC49843.1	Aspfu1|3229	Leptod1|516759	Cadsp1|648036
Arp2	AAF03314.1	Aspfu1|3228	Leptod1|350316	Cadsp1|445556
Abr1	AAF03353.1	Aspfu1|3226	Leptod1|528691	Cadsp1|413886
Abr2	AAF03349.1	Aspfu1|3225	Leptod1|346792	Cadsp1|643922
				:identified by reciprocal best hit
				:Blastp hit, but not best reciprocal best hit

Table S2. Homologous proteins (with JGI ID) involved in melanin synthesis pathways identified in the genomes of *Leptodontium* sp. PMI_412 and *Cadophora* sp. DSE1049 using proteins of *Exophiala dermatitidis* UT8656 following Li et al. (2016).

	Exophiala dermatitidis			
**DHN–melanin pathway**	**as in Li et al. (2016)**	**jgi|ID**	***Leptodontium* sp. PMI_412**	***Cadophora* sp. DSE1049**
**Polyketide synthase (Pks1)**	HMPREF1120-03173	Exode1|3244	Leptod1|526612	Cadsp1|606172
**Abhydrolase (Ayg1)**	HMPREF1120-00377	Exode1|388	Leptod1|423695	Cadsp1|497711
	HMPREF1120-02312	Exode1|2366	Leptod1|423695	Cadsp1|497711
**1,3,6,8-Tetrahydroxynaphthalene reductase (Arp2)**	HMPREF1120-05939	Exode1|6147	Leptod1|350316	Cadsp1|445556
**Scytalone dehydratase (Arp1)**	HMPREF1120-07724	Exode1|7970	Leptod1|380224	Cadsp1|557078
**Fungal pigment MCO (Abr2)**	HMPREF1120-02828	Exode1|2890	Leptod1|346792	Cadsp1|643922
	HMPREF1120-05645	Exode1|5845	Leptod1|346792	Cadsp1|637625
**Fungal ferroxidase (Abr1)**	HMPREF1120-04510	Exode1|4657	Leptod1|107642	Cadsp1|413886
	HMPREF1120-00173	Exode1|177	Leptod1|107642	Cadsp1|413886
	HMPREF1120-01590	Exode1|1625	Leptod1|107642	Cadsp1|413886
	HMPREF1120-03706	Exode1|3803	Leptod1|425821	Cadsp1|570919
	HMPREF1120-04536	Exode1|4683	Leptod1|425821	Cadsp1|570919
**DOPA–melanin pathway**				
**Tyrosinase (melC2)**	HMPREF1120-05316	Exode1|5501	Leptod1|527785	Cadsp1|514369
	HMPREF1120-03345	Exode1|3425	Leptod1|376758	Cadsp1|638399
	HMPREF1120-04514	Exode1|4661	Leptod1|376758	Cadsp1|638399
**Laccase (Lac)**	HMPREF1120-05865	Exode1|6070	Leptod1|525353	Cadsp1|572856
	HMPREF1120-00199	Exode1|203	Leptod1|465495	Cadsp1|18911
	HMPREF1120-08116	Exode1|8373	Leptod1|465495	Cadsp1|572856
	HMPREF1120-08564	Exode1|8840	Leptod1|525179	Cadsp1|658257
	HMPREF1120-04578	Exode1|4726	Leptod1|526126	Cadsp1|658257
	HMPREF1120-02754	Exode1|2815	Leptod1|525179	Cadsp1|658257
**L-tyrosine degradation pathway**				
**Tyrosine aminotransferase (Tat)**	HMPREF1120-02164	Exode1|2216	Leptod1|525973	Cadsp1|477092
**4-Hydroxyphenylpyruvate dioxygenase (hppD)**	HMPREF1120-05584	Exode1|5783	Leptod1|348899	Cadsp1|455878
**Homogentisate dioxygenase (hmgA)**	HMPREF1120-03827	Exode1|3931	Leptod1|379107	Cadsp1|455878
**Fumarylacetoacetate hydrolase (fahA)**	HMPREF1120-03825	Exode1|3929	Leptod1|518059	Cadsp1|455878
**Maleylacetoacetate isomerase (maiA)**	HMPREF1120-03438	Exode1|3525	Leptod1|390379	Cadsp1|455878
				:identified by reciprocal best hit
				:Blastp hit, but not best reciprocal best hit

Table S3. Effect of tricyclazole and kojic acid on fungal growth

Strain	Control	Kojic acid	Tricyclazole	Kojic acid + Tricyclazole
Me07	100 ± 3[a]	101 ± 6[a]	98 ± 4[a]	97 ± 7[a]
Pr30	100 ± 2[a]	90 ± 2[c]	103 ± 2[a]	97 ± 1[b]
Fe06	100 ± 2[c]	112 ± 5[a]	101 ± 1[bc]	109 ± 4[ab]
DSE1049	100 ± 12[b]	115 ± 2[a]	112 ± 4[a]	110 ± 2[a]
Va46	100 ± 9[a]	107 ± 4[a]	102 ± 8[a]	117 ± 4[a]
Pr29	100 ± 4[a]	96 ± 4[a]	95 ± 6[a]	97 ± 4[a]

Mycelia were grown for two weeks on Pachlewski medium amended with 50 µg/mL kojic acid, 10 µg/mL tricyclazole or a combination of both compounds. Values are means ± SE (n=9). Significant differences between treatments ($P < 0.05$, Kruskal-Wallis test) are indicated by different letters.
